# The Histone H4 Lysine 20 Monomethyl Mark, Set by PR-Set7 and Stabilized by L(3)mbt, Is Necessary for Proper Interphase Chromatin Organization

**DOI:** 10.1371/journal.pone.0045321

**Published:** 2012-09-14

**Authors:** Ayako Sakaguchi, Eric Joyce, Tsutomu Aoki, Paul Schedl, Ruth Steward

**Affiliations:** 1 Waksman Institute, Cancer Institute of New Jersey, Rutgers University, Piscataway, New Jersey, United States of America; 2 Department of Molecular Biology and Biochemistry, Rutgers University, Piscataway, New Jersey, United States of America; 3 Department of Molecular Biology, Princeton University, Princeton, New Jersey, United States of America; 4 Department of Genetics, Harvard Medical School, Boston, Massachusetts, United States of America; Ludwig-Maximilians-Universität München, Germany

## Abstract

Drosophila PR-Set7 or SET8 is a histone methyltransferase that specifically monomethylates histone H4 lysine 20 (H4K20). L(3)MBT has been identified as a reader of methylated H4K20. It contains several conserved domains including three MBT repeats binding mono- and dimethylated H4K20 peptides. We find that the depletion of PR-Set7 blocks de novo H4K20me1 resulting in the immediate activation of the DNA damage checkpoint, an increase in the size of interphase nuclei, and drastic reduction of cell viability. L(3)mbt on the other hand stabilizes the monomethyl mark, as L(3)mbt-depleted S2 cells show a reduction of more than 60% of bulk monomethylated H4K20 (H4K20me1) while viability is barely affected. Ploidy and basic chromatin structure show only small changes in PR-Set7-depleted cells, but higher order interphase chromatin organization is significantly affected presumably resulting in the activation of the DNA damage checkpoint. In the absence of any other known functions of *PR-Set7*, the setting of the de novo monomethyl mark appears essential for cell viability in the presence or absence of the DNA damage checkpoint, but once newly assembled chromatin is established the monomethyl mark, protected by L(3)mbt, is dispensable.

## Introduction

Dynamic changes in chromatin structure are directly influenced by the post-translational modifications of the N-terminal histone tails [1], [2]. Specific amino acids within the tails are modified by phosphorylation, ubiquitination, ADP ribosylation, acetylation, and methylation [3]. It has been proposed that distinct histone modifications, on one or more tails, act sequentially or in combination to form a “histone code” that is read by other proteins to bring about distinct events [4], [5].

H4K20 can be mono-, di-, or trimethylated. *Drosophila* PR-Set7 (also known as SET8) is a histone methyltransferase (HMT) that specifically monomethylates H4K20 [6], [7], [8], [9], while di- and trimethylation of the same lysine is controlled by Hmt4-20, also known as Suv4-20 [10], [11]. In vertebrate tissue culture cells, expression of PR-Set7 increases during S phase and peaks at mitosis [12]. The changing levels of PR-Set7 are controlled by its interaction with PCNA which stimulate PR-Set7 ubiquitylation and proteasome-dependent degradation [13], [14], [15].

Detailed analysis of the *PR-Set7* complete loss-of-function phenotype in flies revealed that in neuroblasts the number of both mitotic and S phase cells is reduced, indicating that the cells stop dividing. This analysis also showed that the progression through early mitosis is delayed and that cyclin B protein is strongly reduced [9]. The abnormalities of mitotic progression and the cyclin B protein level were rescued when the DNA damage checkpoint was abolished, indicating that the DNA damage checkpoint is activated when PR-Set7 activity is compromised. However, though the DNA damage checkpoint is activated, there is no evidence of DNA breakage. On the other hand, *PR-Set7* does show defects in chromosome condensation.

Therefore, we proposed that H4K20me1is involved in the maintenance of proper higher order structure of chromatin. Our results were confirmed in human tissue culture cells; when PR-Set7 was depleted in these cells, the DNA damage checkpoint is also activated. However, in contrast to Drosophila, double strand breaks are detected [16], [17]. In these cells, the depletion of PR-Set7 causes S phase delay and alterations in replication fork velocity and origin firing, suggesting that PR-Set7 is involved in S phase progression [16], [17].

A vertebrate PR-Set7 interactor, L3MBTL1, has been identified and PR-Set7 is proposed to enhance its transcriptional repression [18]. L(3)MBT proteins contain several conserved domains, including three MBT repeats [19] that have been shown to bind to mono- and dimethylated histone H4K20 peptides in vitro [20], [21]. L(3)MBT is thought to be a “reader” protein of H4K20me1 (for review see [22] and to promote methylation-dependent chromatin compaction [23].

*l(3)malignant brain tumor* (*l(3)mbt*) was originally identified on the basis of a temperature-sensitive (ts) larval brain cell overgrowth [24]. *l(3)mbt* also has a strong maternal effect phenotype. Embryos from *l(3)mbt* mutant females show abnormalities in cell proliferation [19]. Biochemically L(3)mbt was identified as part of the *Drosophila* Myb complex involved in regulating transcription [25], [26]. Its human ortholog, L3MBTL1, is proposed to function as a negative regulator of E2F target genes such as *c-myc* and *cyclin E1* [23].

To further elucidate the respective functions of *PR-Set7* and *l(3)mbt* we used an RNAi approach in S2 cells. We find that depletion of PR-Set7 inhibits de novo H4K20 monomethylation and results in the activation of the DNA damage checkpoint and a drastic reduction of cell viability. We also detected a low level of abnormal nucleosome compaction in PR-Set7-depleted cells when the ATR checkpoint is abolished. Importantly, these PR-Set7 depleted cells consistently showed an increase in their nuclear diameter, apparently caused by abnormal organization of interphase chromatin. In contrast, the depletion of L(3) mbt reduced the stability of the epigenetic H4K20 monomethyl mark significantly, but it had surprisingly little if any effect on cell viability.

## Materials and Methods

### RNA Interference and Preparation of S2 Cells Extracts

Double-stranded RNAs for *PR-Set7*, *l(3)mbt*, *mei-41*, *grp* and *lacZ* were prepared as previously described [27]. The templates for synthesizing dsRNAs were produced by PCR.

(for *PR-Set7*, 5′-ctaatacgactcactatagggagatgataatggtgcgaagacga-3′ and.

5′-ctaatacgactcactatagggaggcacctcgtcatctagtttta-3′;

for *l(3)mbt*, 5′-ctaatacgactcactatagggagtggcgctccaaaacggtga-3′ and 5′-ctaatacgactcactatagggaggaactgtggtccatagtcc-3′;

for *mei-41*, 5′-ctaatacgactcactatagggaggaaggatatgtggaagttgc-3′ and 5′-ctaatacgactcactatagggagcattccacatcgcatcggaa-3′;

for *grp*, 5′-ctaatacgactcactatagggagcaattacctgcatcagcgtgg-3′ and 5′-ctaatacgactcactatagggagcagcttgcgaagcagcgaa-3′;

for *lacZ*, 5′-ctaatacgactcactatagggagggccaccgatattatttgcc-3′ and 5′-ctaatacgactcactatagggagcacactgaggttttccgc-3′). All primers include the T7 promoter sequence at their 5′ end. Bi-directional transcripts were produced by the Ribomax Large Scale RNA Production System T7 (Promega), using the PCR product as templates. Single-stranded transcripts were annealed by incubating the RNAs at 65°C for 30 min.

*Drosophila* S2 cells [28] were adjusted to grow in Serum-Free medium, SFX-Insect (HyClone). 2.8×10^6^ cells were placed in 4 ml of the medium in 6-well plates and after 30 min 50 µg of dsRNA was added to each well. For preparation of whole cell extracts, 1 ml of cell culture was centrifuged at 1000×g for 1 min and the pellet was lysed in 30 µl of protein sample buffer (62.5 mM Tris pH 6.8, 7% glycerol, 2% SDS, 0.5% BPB, 5% ME). For preparation of Histone extracts, 4 ml of cell culture was centrifuged at 1000×g for 5 min and the cells were washed once in 600 µl of wash buffer (10 mM HEPES pH 7.6, 140 mM NaCl). The pellet was lysed in 300 µl of nuclear extraction buffer (350 mM NaCl, 20 mM HEPES pH 7.6, 0.5 mM EDTA, 1 mM MgCl_2_) and shaken slowly at 4°C for 15 min. The lysate was centrifuged at 12000×g for 1 min and the pellet extracted in 100 µl of 0.25 N HCl for 15 min. The extracts were cleared from the nuclear debris by centrifugation at 12000×g for 1 min, 50 µl of 1 M Tris pH8 and 4x concentrated protein sample buffer were added, and boiled for 15 min.

### Immunoblotting and Over Expression

Immunoblotting was performed as previously described [8]. For histone separation, 14% SDS-PAGE and TTS buffer (100 mM Tris, 100 mM Tricine, 0.1% SDS, pH 8.1–8.5) were used. Rat polyclonal anti-PR-Set7 antibody was diluted 1∶1000. Rabbit polyclonal anti-monomethylated H4K20 (Upstate Biotechnology) was used at 1∶1000 [29]. Rabbit polyclonal anti-histone H3 (Abcam) was used at 1∶15000. Mouse monoclonal anti-lamin D_M_0, ADL67.10 was obtained from the Developmental Studies Hybridoma Bank and used at 1∶2000. Rabbit polyclonal anti-Chk1 (Phospho-Ser^345^; GenScript Corp.) and anti-actin (Sigma) were used at 1∶500 and 1∶1000.

For over expression of L(3)mbt, full length L(3)mbt cDNA was cloned into pMT-FLAG-HA expression vector. 1.2×10^6^ cells were cultured in 4 ml of the medium in 6-well plates for 12 h and then the plasmid was transfected using Effectene (QIAGEN). 18 h after transfection, the expression was induced by addition of 50 µM CuSO_4_. For immunoblotting, mouse monoclonal anti-HA.11 (COVANCE) was used at 1∶1000.

### *In vivo* Histone Methyltransferase Assay

2.0×10^6^ cells were seeded in 4 ml of medium in 6-well plates. After 12 h, 50 µg of dsRNA were added to each well. After 28 h, 0.5 ml of the cell culture was replated in 24-well plates and 8 h later, 15 µl (15 µCi) tritium-labeled SAM (GE Healthcare) was added to each well. After 2 days, cells were replated in 2 ml of medium in 6-well plates and incubated for another 3 days. Histone extracts were prepared from 2 ml of cell culture. The histones were separated by 14% SDS-PAGE and the gel was fixed in fixation buffer (isopropanol: water: acetic acid = 25∶65∶10) for 30 min, shaken slowly in Amplify solution (GE Healthcare) for 30 min and dried at 80°C for 2.5 h. The autoradiograph was developed after 10-day incubation at −80°C.

### Cell Viability and DNA Double Strand Break Assays

1.6–2.8×10^6^ cells were placed in 4 ml of the medium in 6-well plates and dsRNA was added as described above (day 0). The number of cells was counted from day 0 to day 10 and the average ratio of each value to the values of *lacZ* knockdown cells was calculated. Each time the number of the cells reached 1×10^7^, the cell culture was diluted by 25% to avoid saturation density. For double strand break assay the cells were harvested 4 days after addition of dsRNA and stained with Rabbit polyclonal anti–PH2Av (Ser139) antibody (Upstate Biotechnology) diluted 1∶500. Doulbe strand breaks were induced by exposing the cells to 10 Gy for 10 minutes.

### FACS Analysis

To perform FACS analysis 1 ml of cell culture was centrifuged at 800×g for 5 min. The pelleted cells were washed in 10 ml of phosphate-buffered saline (PBS) and resuspended in 500 µl of PBS with 0.1% Triton-X containing 25 µg/ml propidium iodide. FACS was performed with Cytomics FC500 Flow Cytometer (Coulter).

### Micrococcal Nuclease Digestion

RNAi treatment was performed as described above. S2 cells were harvested after 6 days of treatment. Nuclei were isolated and digested with micrococcal nuclease as described earlier [30], except that 0.1 unit of micrococcal nuclease (Sigma) was added per 1 O.D. (260 nm). Digested DNAs were separated on 1% agarose gels and transferred to nitrocellulose filters. The 5S rRNA plasmid 22A8 [31] was labeled using Ready-To-Go DNA Labeling Beads (GE Healthcare Life Sciences) and hybridized.

### FISH Analysis

Our FISH protocol has been previously described [32]. S2 cells were treated with dsRNA for 3 days. Oligo probes for the 359 and dodeca heterochromatic repeats were synthesized with a 5′ Cy5 and Alexa488 fluorescent dye, respectively, from Integrated DNA Technologies (IDT). The sequences are as follows: Cy5-359: Cy5-gggatcgttagcactggtaattagctgc and Alexa488-dodeca: Alexa488- ACGGGACCAGTACGG. DNA probes to 28B and 69C were synthesized according to standard protocols. P1 plasmids (Berkeley Drosophila Genome Project) containing cloned Drosophila genomic DNA corresponding to chromosomal regions 28B1–28B2 (abbreviated as 28B; DS01529) and 69C2–69C8 (abbreviated as 69C; DS02752; [33]) were synthesized and labeled by nick translation/direct labeling (Vysis) following the manufacturer’s protocol. [32], [34].

FISH images were collected using an Olympus IX81 fluorescence microscope with a 60X, N.A. 1.35 lens. Nuclei were imaged by collecting optical sections through the entire nucleus and the analysis of the images was performed by examining one section at a time. The images are from a single section. The number of FISH signals per nucleus was counted manually and the nuclear diameters were measured using Olympus cellSens software.

## Results

**Figure 1:**
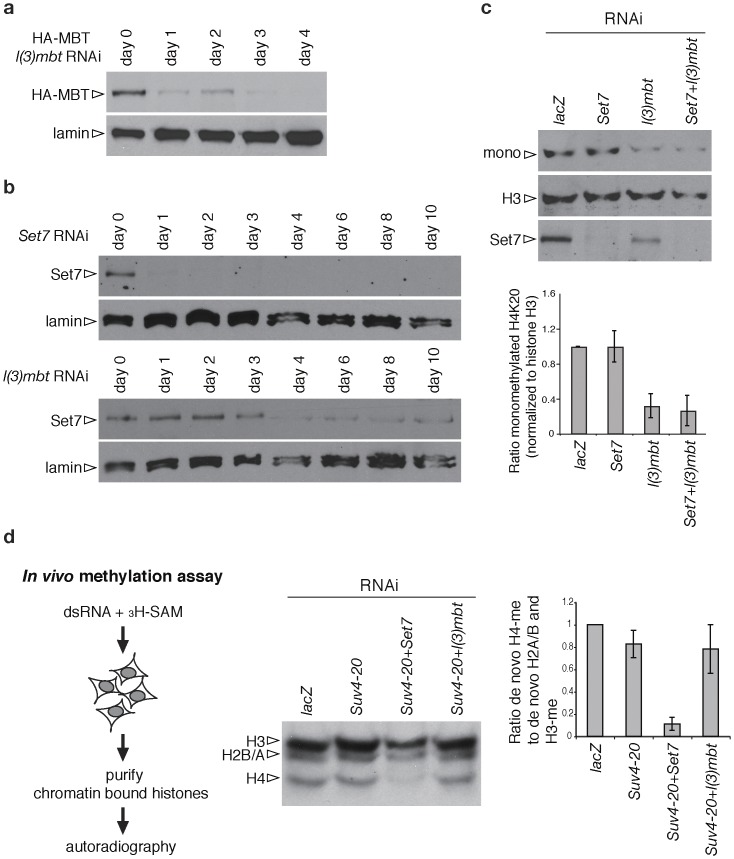
PR-Set7 functions independently of L(3)MBT in controlling the H4K20 monomethyl mark. **a**, HA tagged full length L(3)MBT (HA-MBT) was expressed in S2 cells and monitored after RNAi against *l(3)mbt*. **b,** PR-Set7 levels in *PR-Set7* and *l(3)mbt* knockdown S2 cells. **c**, Blots of extracts from S2 cells treated for five days with RNAi, probed with anti-mono Histone H4K20 (mono), anti-histone H3 (H3) and anti-Set7 antibodies (Set7). The graph shows the average ratio of each value to the values of *lacZ* cells. The value of monomethylated H4K20 was normalized to the value of histone H3. **d**, De novo methylation was assessed by supplying tritium-labeled SAM, to S2 cells treated with different RNAis. The graph shows the average ratio of de novo histone H4 methylation to total *de novo* histone methylation of H2A, H2B and H3. Error bars for both graphs show two SDs (n = 3). The knockdown of *l(3)mbt* does not affect the level of tritium labeling on histone H4, showing that newly monomehtylated H4K20 is stable for duration of the experiment even in the absence of L(3)MBT.

We examined the importance of both *PR-Set7* and *l(3)mbt* in monomethylation of H4K20 using an RNAi approach in S2 cells. To establish that *l(3)mbt* RNAi treatment reduces the level of the protein we expressed HA tagged full length L(3)mbt and monitored HA-L(3)mbt by western blots. We observed significant reduction of HA-L(3)mbt from day one onward (Figure 1a), indicating that the level of endogenous L(3)mbt is also likely to be strongly diminished. We next monitored PR-Set7 levels for ten days in *PR-Set7* knockdown cells and found that the protein is strongly reduced within one day of RNAi treatment (Figure 1b). In *l(3)mbt* knockdown cells PR-Set7 levels also noticeably decreased on day 4 and stayed at this low level for the duration of our experiment. In contrast to what would be expected based on published results [18], other transcripts are also reduced, indicating that reducing L(3)mbt levels may affect transcription of many genes (Figure S1 and see [35]).

We next determined the effect of lowering *PR-Set7*, *l(3)mbt*, *PR-Set7 l(3)mbt* and, as control, *lacZ*, on the level of H4K20me1 five days after RNAi (Figure 1c). In *PR-Set7* no change in H4K20me1 was observed on western blots even though PR-Set7 is strongly reduced. Likely several factors contribute to this finding. First, when PR-Set7 is depleted, activation of the DNA damage checkpoint arrests cell cycle progression and presumably also limits the synthesis of new histones. Second, a pool of unincorporated H4K20me1 protein may exist in S2 cells as our previous *in vivo* data show the presence of unincorporated H4K20me1 in early embryos [36]. Further, H4K20me1 is stable over several cell generations in flies [8], [9]. In contrast in *l(3)mbt* and in *PR-Set7 l(3)mbt* cells H4K20me1 is reduced to 32% and 27% of control (*lacZ* RNAi) levels respectively when the value is normalized to the level of histone H3 (Fig. 1c). Similar results were obtained in time course experiments (see Fig. S2). These results indicate that only the depletion of L(3)mbt reduces the H4K20 monomethyl mark significantly.

Since our western experiments did not show that H4K20me1 levels are dependent on *PR-Set7*, we sought an alternative means for connecting this histone modification to *PR-Set7* activity. For this purpose we measured HMT activity on histones in cells in an *in-vivo* assay (Figure 1d). We followed de novo methylation of each histone 6 days after RNAi by growing the cells in the presence of tritium-labeled HMT substrate, SAM (*s*-Adenosyl-_L_-[methyl-^3^H]methionine). In *Drosophila*, di- and trimethylation of H4K20 is controlled by a single HMT, Suv4-20 [11]. Hence, we assayed de novo methylation in cells treated with *Suv4-20 PR-Set7* and *Suv4-20 l(3)mbt*, and as control, *Suv4-*20, and *lacZ* RNAi, and compared the ratio of de novo histone H4 methylation (de novo H4-me) to total de novo histone methylation of H2A, H2B and H3. The depletion of Suv4-20 results in 17% reduction of de novo H4-me. In the *Suv4-20 PR-Set7* cells de novo H4-me decreased by ∼90% of the control level, indicating that knockdown of *PR-Set7* almost completely eliminated HMT activity on histone H4. However, the double knockdown of *Suv4-20* and *l(3)mbt* resulted in only 22% reduction of de novo H4-me, a reduction statistically identical to the result for *Suv4-20* cells (p = 0.56, t-test). These results show that *PR-Set7* functions independently of *l(3)mbt* in monomethylating H4K20, and that *l(3)mbt* must have some other function resulting in the protection or stabilization of existing H4K20me1.

**Figure 2:**
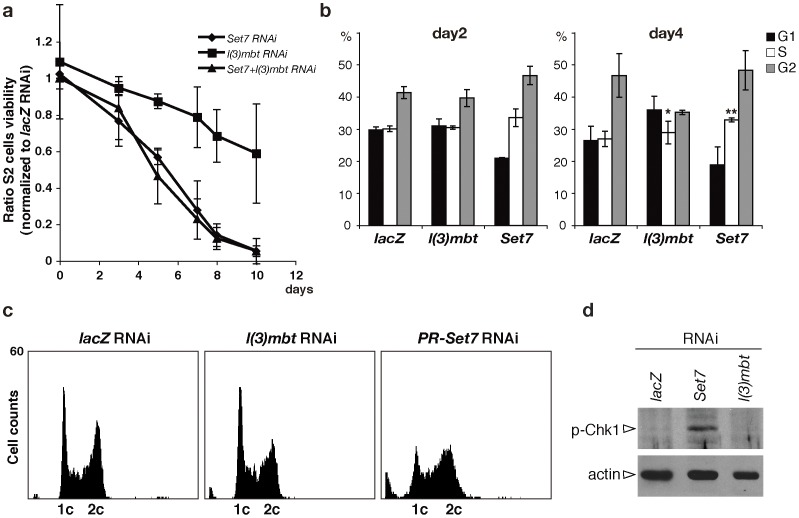
De novo monomethylation of H4K20 is essential for S2 cells. a , Cell viability. The graph shows the average ratio of each cell count to that of *lacZ* RNAi cells (n≧3). **b**, Cell cycle profiles. The number of S phase cells on day 4 is significantly increased in *PR-Set7* cells (**; p = 0.022, t-Test) and not significantly changed in *l(3)mbt* cells (*; p = 0.336). Each error bar is two SDs. **c**, DNA contents on day 4. **d**, Blots of extracts from S2 cells after four days of RNAi treatment, probed with anti-phosphorylated Chk1 (p-Chk1).

Next we compared the effects of depleting PR-Set7 and L(3)mbt on cell growth and viability. Depletion of L(3)mbt cells has little if any effect on cell viability. Such cells continue to grow at a rate comparable to that of the *lacZ-*knock down control over a period of more than a week, even though the monomethyl mark is reduced to 40% after 5 days. In contrast, depletion of PR-Set7 reduces cell viability drastically over a ten-day period (Figure 2a). These results seem to indicate that in the tissue culture context, de novo H4K20me1 is essential for continued growth unless PR-Set7 has any, so far elusive, additional functions. On the other hand L(3)mbt and at least 60% of the epigenetic mark are largely dispensable.

Since L(3)mbt is thought to specifically recognize and thus potentially monitor nucleosomes containing H4K20me1 [18], [23], [25], [26], the growth arrest evident in *PR-Set7* cells could be mediated by an L(3)mbt-dependent signal. If this were the case, then reducing L(3)mbt activity in the *PR-Set7* knockdown should relieve the growth defects. As shown in Figure 2a, this prediction is incorrect; depletion of both PR-Set7 and L(3)mbt arrests growth with approximately the same kinetics as PR-Set7 depletion alone. This finding indicates that the growth defects induced by PR-Set7 depletion are not mediated by an L(3)mbt dependent recognition of H4K20me1.

To better assess cell-cycle defects in knockdown cells, we used FACS analysis to profile the cell cycle (Figure 2b, c). In *PR-Set7,* a change in the number of G1 and G2 cells is already observed on day 1 (data not shown). By day 4 the number of S phase cells is significantly increased (p = 0.022, t-Test, Fig. 2b), similar to what is observed in PR-Set7-depleted human cells [16], [17]. On day 4 the histogram of DNA content of *PR-Set7* cells shows that the two peaks at 1c (2N) and 2c (4N) are lower and broader (Figure 2c). The number of cells categorized as sub-G1 or polyploid is also increased, suggesting that in many cells the DNA content is abnormal. As would be expected from the minimal effect of the *l(3)mbt* knockdown on cell growth, the number of S phase cells and the histogram of these cells are not significantly different from those of control cells.

**Figure 3:**
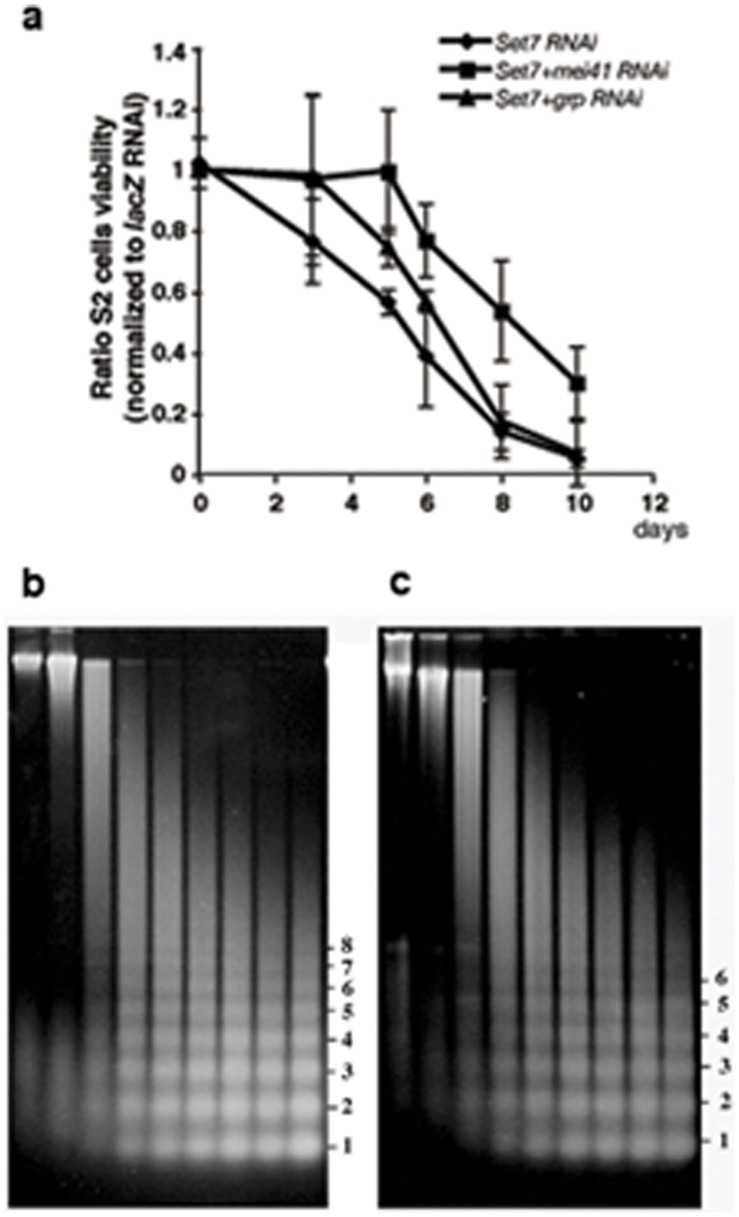
Cell viability and nucleosome analysis in normal and *PR-Set7 mei-41* cells. The graph shows the average ratio of each cell count to that of *lacZ* RNAi cells (n≧3). The knockdown of either *mei-41* (Drosophila *ATR*) or *grp* (*Chk1*) disrupts the DNA damage checkpoint and therefore rescues the lethality of the *PR-Set7* cells partially. Nucleosomal ladders from *mei-41* (**b**), and *PR-Set7 mei-41* (**c**) knockdown cells. Nuclei were subjected to micrococcal nuclease digestion for the times indicated, separated electrophoretically.

We previously proposed that H4K20me1 is involved in the maintenance of proper chromatin structure because in *PR-Set7* mutant brains the DNA damage checkpoint is activated but DNA double-strand breaks are not increased [9]. To confirm that the depletion of de novo H4K20me1 causes the checkpoint activation, we looked for phosphorylated Chk1, a marker of checkpoint activation [37] and detected the phosphorylated form only in *PR-Set7* (Figure 2d). We next asked whether the cell growth defects induced by knocking down *PR-Set7* is rescued by simultaneously knocking down the DNA damage checkpoint gene *mei-41*. Whereas knocking down *PR-Set7* alone arrests cell growth and division after about 4 or 5 days, cells simultaneously knocked down for *PR-Set7* and either *mei-41* or the Drosophila checkpoint kinase *grp* continue to grow for 6 or 7 days before growth shows evidence of slowing (Figure 3A). We previously found that loss of PR-Set7 in Drosophila larvae did not result in DNA double strand breaks as judged by staining for anti–phosphorylated histone H2Av [9]. We did the same experiments on control and PR-Set7 depleted S2 cells and found that that even though the DNA damage checkpoint is activated, we could not observe double strand breaks by anti–phosphorylated histone H2Av (Table S1). Taken together these results suggest that the growth arrest in the single *PR-Set7* knockdown cells is due to the activation of the DNA damage checkpoint, while another unknown mechanism must be responsible for the reduction in growth rate seen later on in the *PR-Set7 mei-41* or *PR-Set7 grp* double knockdown cells.

In the *Pr-Set7* knockdown experiments in vertebrates, cells tend to accumulate in S phase [16], [17]. It is possible that abnormalities in chromatin organization could occur because de novo H4K20 methylation is tightly coupled to the process of nucleosome assembly, and therefore in the absence of this methylation mark DNA synthesis and de novo nucleosome assembly could become uncoupled. In this scenario, when *Pr-Set7* activity is compromised, DNA replication would continue without properly assembling chromatin on the newly replicated DNA. The presence of stretches of abnormally packaged DNA would then activate the DNA damage checkpoint.

We therefore examined the effects of reducing PR-Set7 on chromatin architecture in double knockdown (*PR-Set7;mei41).* Figures 3b and c show the nucleosomal ladders produced by digestion of nuclei prepared from either *mei-41* (b) or *PR-Set7;mei-41* (c) knockdown cells. The most striking finding is that the nucleosomal ladder remains largely intact in the *PR-Set7;mei-41* knockdown cells, and that there is no evidence of extensive regions of DNA that lack nucleosomes. Since the double knockdown cells have divided once or twice under conditions that would have induced a cell cycle arrest in the single knockdown cells, it would appear that *PR-Set7* is not required to sustain the process of de novo nucleosome assembly. While no gross disruptions in global nucleosome organization are observed, some differences between the double knockdown and the single knockdown control were evident in this and other experiments suggestive of differences in nucleosome packing. The double knockdown appears to be more sensitive to micrococcal nuclease digestion (Fig. 3, compare corresponding lanes in panels b and c). Larger polynucleosomes (4–8 mers) are present in lower yield and/or appear less distinct (e.g., 5 & 6) than in the control knockdown. This increased sensitivity could indicate that nucleosome arrays/bulk chromatin may not be as tightly compacted on average in the double knockdown compared to the control cells.

**Figure 4:**
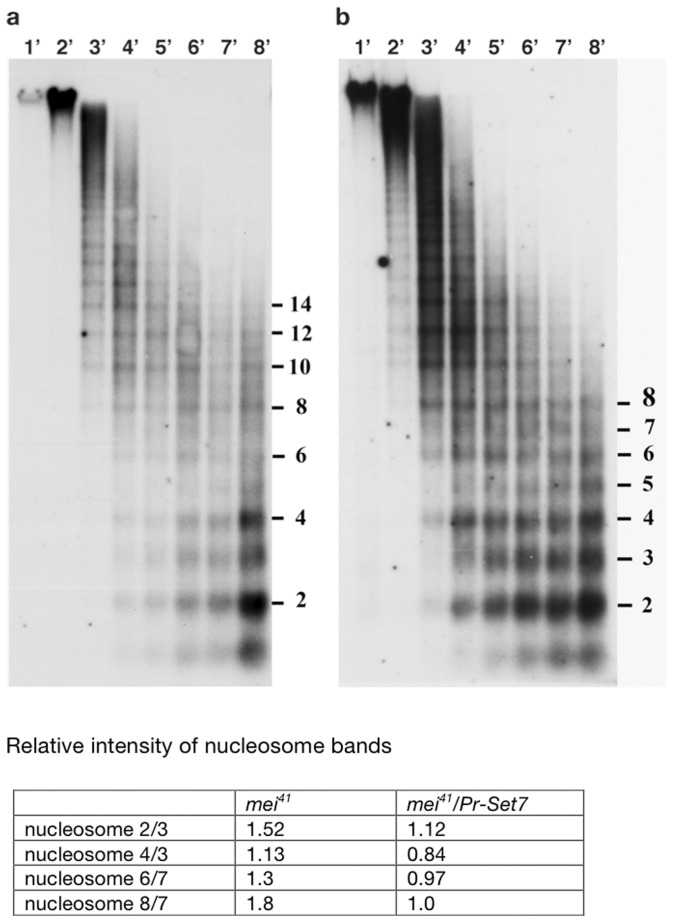
PR-Set7-depleted cells show abnormal nucleosome spacing. Nuclei isolated from control *mei-41* cells (**a**), and *PR-Set7 mei-41* (**b**), were subjected to micrococcal nuclease digestion for the indicated times, and separated electrophoretically. The DNA was transferred to nitrocellulose and hybridized with a ^32^P-labeled 5S probe [31]. The intensity of the radioactivity was measured and calculated as a ratio of the even numbered nucleosome divided by the odd one; e.g. ratio of dinucleosome/trinucleosome, tetranucleosome/trinucleosome etc.

To confirm these findings we examined the chromatin organization of the ∼100 tandemly repeated 5 s rRNA genes. Previous studies show that the two nucleosomes associated with the 375 bp 5 s RNA gene + spacer are positioned relative to the underlying DNA sequence [31], [38]. As a consequence, MNase preferentially generates multiples of two nucleosomes, while fragments corresponding to an odd number of nucleosomes are considerably less frequent (see Fig. 4a). The 5 s repeat also contains sequences that are preferentially cleaved by micrococcal nuclease in naked DNA digests. Normally these sequences are incorporated into nucleosomal DNA and are comparatively resistant to micrococcal cleavage; however, if nucleosomes were depleted in some of the 5 s rDNA repeats, these sites would be exposed and generate fragments of non-nucleosomal lengths. Figure 4b shows the characteristic di-nucleosomal ladder of 5 s rDNA repeats generated by micrococcal nuclease digests of chromatin from *PR-Set7; mei-41* double knockdown cells, and there is no evidence of enhanced cleavage at naked DNA sites. This result supports the main conclusion drawn from the MNase cleavage pattern in bulk chromatin, namely that de novo nucleosome assembly can proceed in the absence of *PR-Set7* activity under conditions in which activation of the DNA damage checkpoint is blocked. Additionally, as was evident in the bulk chromatin digest, there appear to be some abnormalities in the 5 s rDNA digestion pattern that are suggestive of differences in nucleosome compaction. The 5 s chromatin in the double knockdown cells is more sensitive to MNase digestion and large oligonucleosomes appear earlier in the digestion and are lost more rapidly. The yield of odd numbered bands (3, 5 and 7 nucleosomes) also is visibly elevated compared to the control. Both the increased sensitivity of 5 s chromatin and the increase in the yield of odd number bands (particularly the trinucleosome) was seen in several experiments.

**Figure 5:**
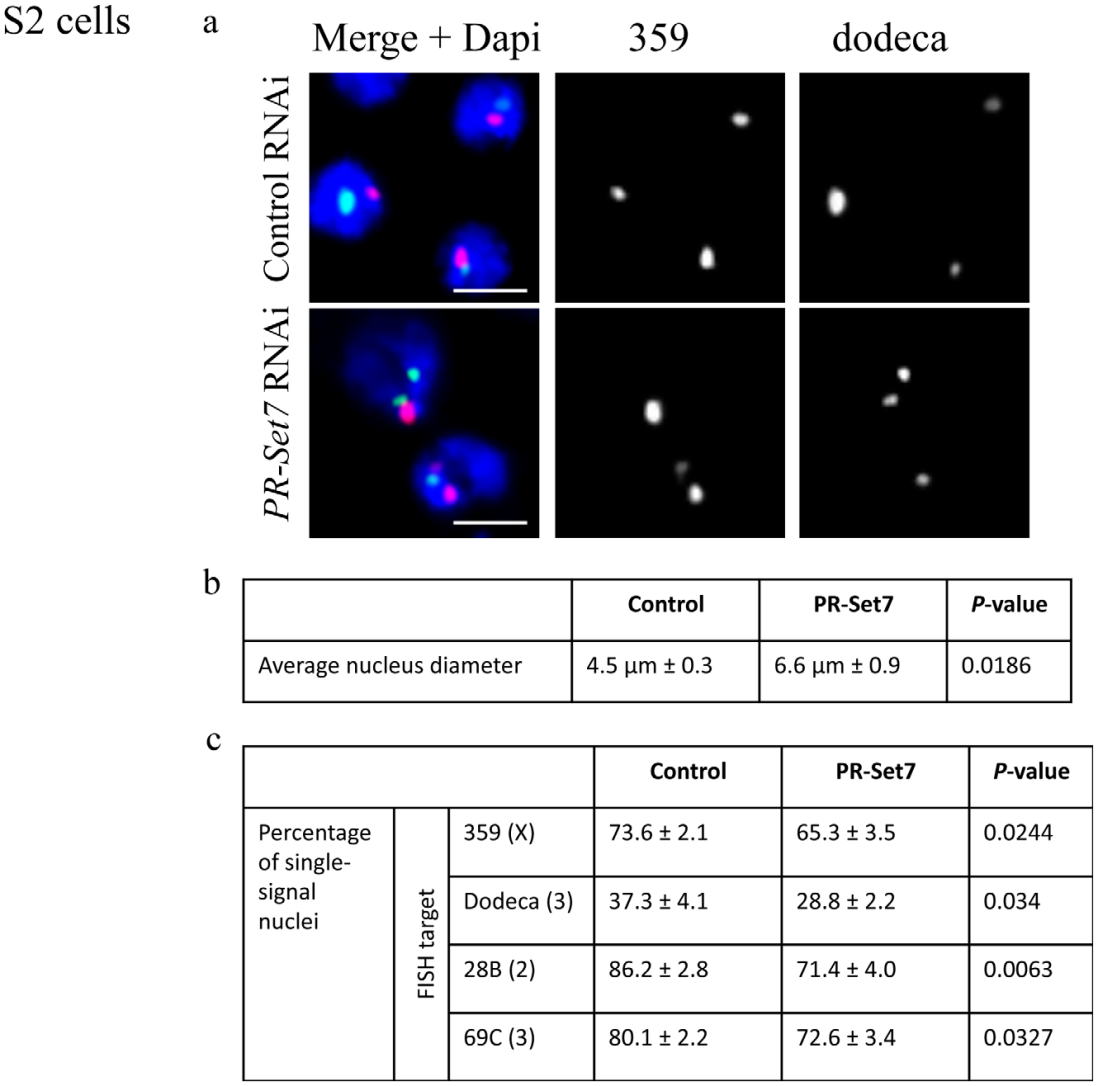
PR-Set7-depleted cells show disrupted nuclear organization. (**a**) Representative images of PR-Set7-depleted S2 cells that exhibit larger nuclei and increased number of FISH signals per nucleus. Scale bars are 5 µm. (**b**) The average nuclear diameter is increased as compared to control cells (*lacZ* RNAi). (**c**) dsRNA directed against PR-Set7 decreases the number of nuclei with a single FISH signal when targeting peri-centromeric heterochromatic regions (359 on X, red; dodeca on 3^rd^, green) or euchromatic regions (28B on 2^nd^; 69C on 3^rd^). A minimum number of 100 nuclei were counted for each of three replicate tests. *P*-values were calculated using an unpaired *t* test.

Though the experiments reported above clearly demonstrate that *PR-Set7* is not needed for de novo nucleosome assembly, the fact that overall micrococcal nuclease sensitivity appeared to be elevated, albeit only slightly, was intriguing when considered in the context of previous studies on larval brains in *PR-Set7* mutants [9]. In these studies, we found that interphase nuclei in the *PR-Set7* mutants are enlarged compared to nuclei in wild type brains. Indeed, as was observed in the fly, about 20% of PR-Set7-depleted tissue culture cells had nuclear volumes at or above the 95^th^ percentile of control knockdown cells. The average nuclear diameter in PR-Set7-depleted cells was 6.6 µm compared to 4.5 µm in controls (Fig. 5). We considered the possibility that this phenotype could reflect polyploid cells that arise due to the role of PR-Set7 in mitosis. However, the modest increase in polyploidy observed in our FACS analysis cannot explain the significant proportion of cells with this phenotype.

An alternative explanation is that the enlarged nuclei are caused by an alteration in high order chromatin compaction or organization. We reasoned that if higher-order chromatin folding was perturbed in PR-Set7-depleted cells, the spatial integrity of large chromosomal regions might be altered relative to control cells. To test this possibility, we performed DNA-FISH in male-derived S2 cultured cells [39] that contain two X chromosomes due to tetraploidy of the cell line [40]–[41]. Following *PR-Set7* knockdown, we observed an increase in the number of FISH signals targeting the ∼11 Mb 359-bp repeat, located in the pericentromeric heterochromatin of the X chromosome; only 65% of PR-Set7-depleted cells exhibited a single FISH signal as compared to 74% in control cells (Fig. 5; p<0.0244). The increase in the number of FISH signals was not unique to the X chromosome and was also observed when FISH targeted the dodeca satellite repeat on the 3^rd^ chromosome as well as two euchromatic regions, 28B and 69C, on the 2^nd^ and 3^rd^ chromosomes, respectively (Fig. 5). Similar results were obtained in a recent screen that showed that dsRNAs targeting PR-Set7 caused a disruption in MSL staining, a complex important for dosage compensation, and an increase in the number of FISH signals in Drosophila cultured cells, which the authors suggests is due to a disruption in somatic homolog pairing [42].

We propose that increased FISH signals could be explained by defects in chromatin condensation, chromosome breakage, or separation of sister chromatids or homologs, each of which would be consistent with a role for PR-Set7 in higher-order chromatin organization during interphase in Drosophila cells. This would suggest that H4K20me1 is essential for chromatin packaging both during interphase and mitosis [9], [16], [43]. The failure to fully condense chromatin could also explain our nucleosome results. It is likely that the differences between normal and PR-Set7 nucleosome positioning are lost when chromatin that is not properly condensed is digested.

## Discussion

In our studies we established that PR-Set7 sets the H4K20 monomethyl mark in vivo and that at least in S2 cells K20 is the only amino acid that is methylated. We further found that depleting PR-Set7 in Drosophila S2 cells leads to the activation of the DNA damage checkpoint and within about 10 days to cell death. When the DNA damage checkpoint is abrogated by double knock-down of *PR-Set7* and the checkpoint genes *mei-41* or *grp,* the half/life of the cells is increased by 1 to two days, but ultimately the cells still die, suggesting that whatever is perturbed in the absence of PR-Set7 cannot be repaired. We did not observe double strand breaks when staining for anti–phosphorylated histone H2A. This does not agree with results observed in vertebrate cells [17] [16] and may be because, in Drosophila, H2Av is not phosphorylated in the absence of H4K20me1 or the specific epitope is obscured. Alternatively, double strand breaks may not exist and the checkpoint is activated because of abnormal chromatin organization or because protein complexes are not removed in a timely manner as observed in *Saccharomyces cerevisiae* [44].

In this context it is interesting to note that in vertebrates H4K20 me2 is implicated in double strand break repair [45]. Because H4K20me1 is the likely substrate for Suv4-20H1 and H2, the di- and trimethyltransferases, an additional link between H4K20 methylation and double strand breaks seems to exist. However, besides potentially setting the monomethyl mark at double strand breaks, PR-Set7 would have to have additional functions, because in both flies and vertebrates PR-Set7 mutants have a substantially stronger phenotype than the loss of the Suv4-20 enzymes [46] [11].

The increase in nuclear volume, together with the changes in the number of FISH signals per nucleus observed in interphase cells following PR-Set7 RNAi would be consistent with a role for PR-Set7 in chromosome compaction and higher-order chromatin organization ( [9], [16], [43], Bateman, 2012 #179). Interestingly, mass spectrometry experiments show that the H4K20 monomethyl mark is set at the G2/M transition well after newly synthesized histone H4 is incorporated into chromatin in S phase [47]. These findings suggest that the abnormalities in chromosome compaction and organization evident in interphase nuclei might be due to defects arising during the G2/M transition. Consistent with this possibility, cells depleted for only Pr-Set7 appear to arrest mostly in early mitosis. But unlike what is observed in larval brains, there is also a subset of cells that arrest in S phase [9]; these may represent cells that despite the abnormalities in higher order chromatin organization are able to continue through the cell cycle until a checkpoint is activated during S. The discrepancy between the brain and tissue culture cells may be a reflection of differences in their cell cycle and developmental potential.

Results from several laboratories suggest that *PR-Set7* function is coupled to DNA replication based on its targeting to the dividing fork via its interaction with PCNA [17] [15]. Our findings indicate that while abnormalities in chromatin organization and compaction appear to accumulate after growth without *Pr-Set7* activity, these defects are inconsistent with massive disruptions in *de novo* nucleosome assembly during replication [48]. Instead, the DNA damage checkpoint activation must arise from more subtle abnormalities in chromatin or DNA structure.

As for the *l(3)mbt*, its functional requirement does not appear to overlap with that of *PR-Set7*, neither in tissue culture as shown here, nor in flies. In larvae the loss of *l(2)mbt* results in an expansion of the neuroblast pool and subsequent tumorous overgrowth of the optic lobe [49] while in *PR-Set7* mutants the cell cycle of neuroblasts arrests in early mitosis resulting in fewer cells [9]. PR-Set7 is essential for de novo methylation of H4K20. While the loss of H4K20me1 could occur either because in the absence of L(3)mbt protection the H4K20me1 is lost, or it could be a secondary effect. Consistent with the latter explanation, recent results show that L(3)mbt binds to DNA boundary elements and affects the level of transcription of Salvador-Wart-Hippo pathway genes both positively and negatively [42]. That L(3)mbt possibly controls expression of many genes is also supported by our observation that the transcription level of all genes we tested was reduced compared to wild type (Fig. 1S).

## Supporting Information

Figure S1 **Transcriptional levels of several genes measured by quantitative real-time PCR five days afterRNAi treatment against** ***PR-Set7*** **and** ***l(3)mbt*** **.** Each value is normalized to the level of *rp-49*. The graph shows the average ratio of each value of *PR-Set7* and *l(3)mbt* knockdown cells to the values of *lacZ* knockdown cells (control). Error bars show two SDs (n≧3). The asterisk means that the value was not detected.
(TIFF)

Figure S2 **Time-course experiment to detect the reduction of monomethylated H4K20 in** ***PR-Set7*** **and** ***l(3)mbt*** **knockdwon cells.** The number of cells was counted at each point and the number of cell divisions was calculated. The intensity of the bands was quantified by ImageJ, and the value of monomethylated H4K20 (mono) was normalized to the values of both histone H3 and *lacZ* knockdown cells (control). Cells were plated at two densities (1.6×10 and 2.8×10 ). Two examples of western blots are shown (a and b). In the *l(3)mbt* cells H4K20 monomethylation is reduced to around 40% of controls after four divisions. In the *PR-Set7* cells monomethylation is reduced by at most 21%, suggesting that the slow growth of *PR-Set7* cells (see Figure 2a) is not the reason why we failed to detect reduction of the monomethylation.
(TIF)

Table S1 **The number of double strand breaks is not increased in PR-Set7 depleted cells. (>750 cells were counted for each sample).**
(PDF)
