# Supplementary figures and images for: The Histone H4 Lysine 20 Monomethyl Mark, Set by PR-Set7 and Stabilized by L(3)mbt, Is Necessary for Proper Interphase Chromatin Organization
Source: PLoS One. 2012 Sep 14;7(9):e45321. doi: 10.1371/journal.pone.0045321 (PMC3443217; doi:10.1371/journal.pone.0045321)

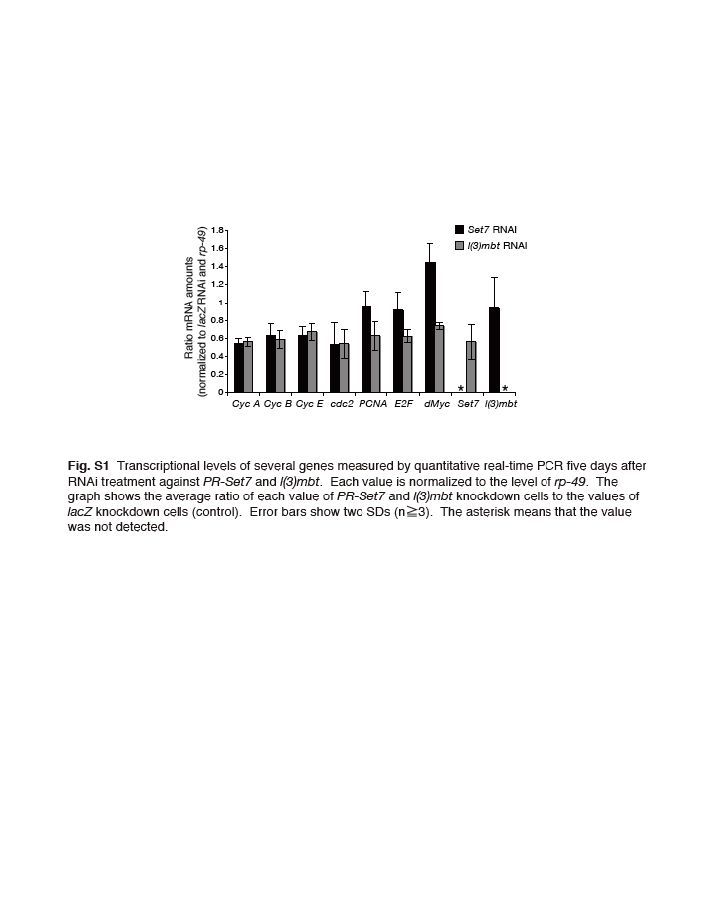

Supplement: Figure S1 — Transcriptional levels of several genes measured by quantitative real-time PCR five days afterRNAi treatment against PR-Set7 and l(3)mbt . Each value is normalized to the level of rp-49. The graph shows the average ratio of each value of PR-Set7 and l(3)mbt knockdown cells to the values of lacZ knockdown cells (control). Error bars show two SDs (n≧3). The asterisk means that the value was not detected. (TIFF) [file pone.0045321.s001.tif]

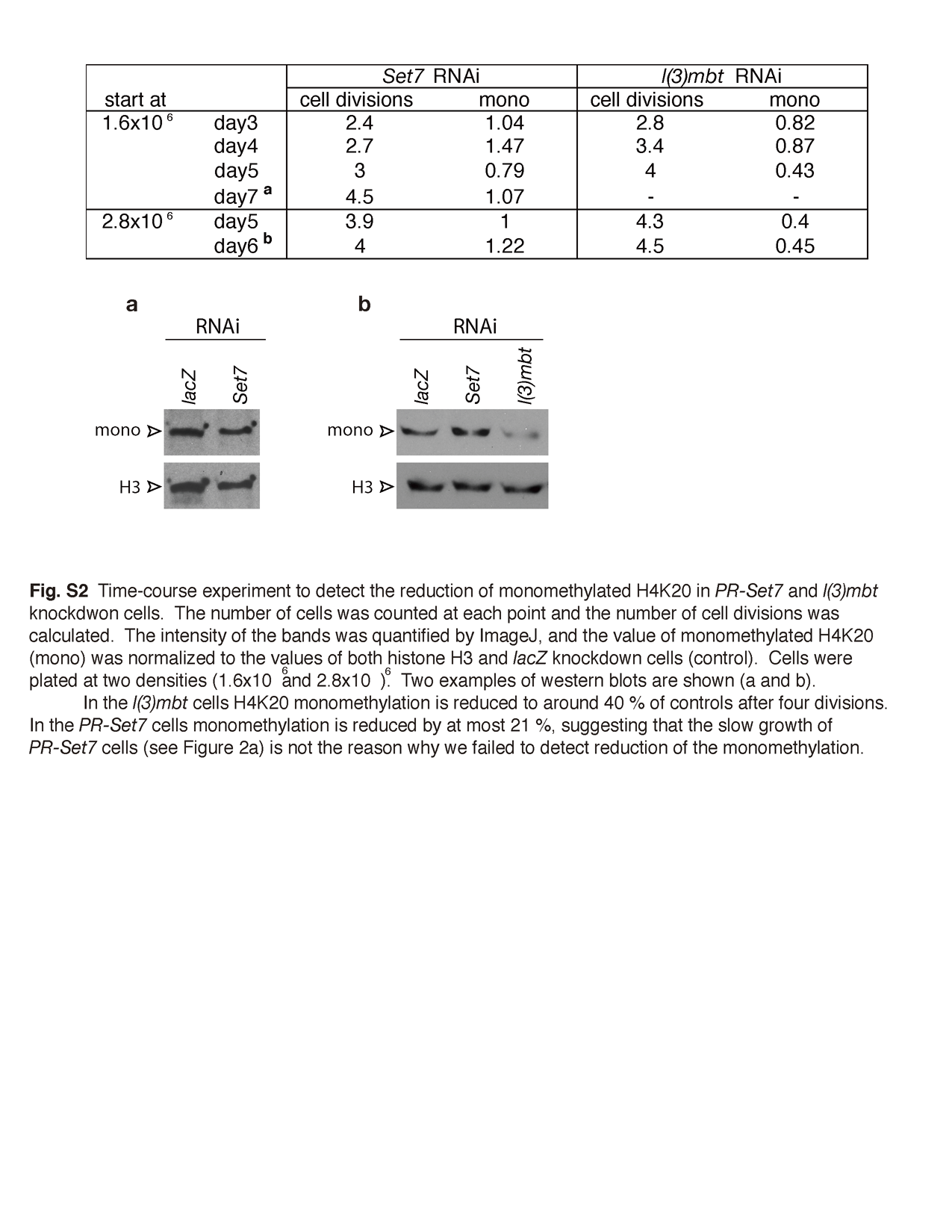

Supplement: Figure S2 — Time-course experiment to detect the reduction of monomethylated H4K20 in PR-Set7 and l(3)mbt knockdwon cells. The number of cells was counted at each point and the number of cell divisions was calculated. The intensity of the bands was quantified by ImageJ, and the value of monomethylated H4K20 (mono) was normalized to the values of both histone H3 and lacZ knockdown cells (control). Cells were plated at two densities (1.6×10 and 2.8×10 ). Two examples of western blots are shown (a and b). In the l(3)mbt cells H4K20 monomethylation is reduced to around 40% of controls after four divisions. In the PR-Set7 cells monomethylation is reduced by at most 21%, suggesting that the slow growth of PR-Set7 cells (see Figure 2a) is not the reason why we failed to detect reduction of the monomethylation. (TIF) [file pone.0045321.s002.tif]
