# Supplementary material for: The Histone H4 Lysine 20 Monomethyl Mark, Set by PR-Set7 and Stabilized by L(3)mbt, Is Necessary for Proper Interphase Chromatin Organization
Source: PLoS One. 2012 Sep 14;7(9):e45321. doi: 10.1371/journal.pone.0045321 (PMC3443217; doi:10.1371/journal.pone.0045321)
Supplement: Table S1 — The number of double strand breaks is not increased in PR-Set7 depleted cells. (>750 cells were counted for each sample). (PDF) [file pone.0045321.s003.pdf]

		PH2Av pos.	% positive
non-irr.	*WT*	80	10
	PR-Set7	113	13
irr.	*WT*	686	88
	PR-Set7	696	90

Table S1. PH2Av staining. > 750 cells were counted
